# Thermal resistivity and hydrodynamics of the degenerate electron fluid in antimony

**DOI:** 10.1038/s41467-020-20420-9

**Published:** 2021-01-08

**Authors:** Alexandre Jaoui, Benoît Fauqué, Kamran Behnia

**Affiliations:** 1 JEIP, USR 3573 CNRS, Collège de France, PSL Research University, 11, Place Marcelin Berthelot, Paris, Cedex 05, 75231 France; 2 Laboratoire de Physique et Etude des Matériaux (CNRS/UPMC), Ecole Supérieure de Physique et de Chimie Industrielles, 10 Rue Vauquelin, Paris, 75005 France

**Keywords:** Electronic properties and materials, Quantum fluids and solids

## Abstract

Detecting hydrodynamic fingerprints in the flow of electrons in solids constitutes a dynamic field of investigation in contemporary condensed matter physics. Most attention has been focused on the regime near the degeneracy temperature when the thermal velocity can present a spatially modulated profile. Here, we report on the observation of a hydrodynamic feature in the flow of quasi-ballistic degenerate electrons in bulk antimony. By scrutinizing the temperature dependence of thermal and electric resistivities, we detect a size-dependent departure from the Wiedemann-Franz law, unexpected in the momentum-relaxing picture of transport. This observation finds a natural explanation in the hydrodynamic picture, where upon warming, momentum-conserving collisions reduce quadratically in temperature both viscosity and thermal diffusivity. This effect has been established theoretically and experimentally in normal-state liquid ^3^He. The comparison of electrons in antimony and fermions in ^3^He paves the way to a quantification of momentum-conserving fermion-fermion collision rate in different Fermi liquids.

## Abstract (web summary)

Viscous fermionic flow appears in liquid helium but rarely appears in metallic solid. Here, Jaoui et al. report a T-square thermal resistivity due to momentum conserving electronic scattering in semi-metallic antimony, which is in agreement with the hydrodynamic scenario.

## Introduction

The possibility of viscous electronic flow, suggested long ago by Gurzhi^1^, has attracted a lot of attention recently ^2–4^. When momentum-conserving (MC) collisions among electrons outweigh scattering by boundaries as well as various momentum-relaxing (MR) collisions, the quasiparticle (QP) flow profile is expected to change. In this case, momentum and energy of the QPs will be redistributed over a length much shorter than the resistive mean free path. As a consequence, the further away the electron is from the boundaries, the hardest the MC collisions will make it for the QP to make its way to the boundaries of the system. If boundary scattering becomes also more frequent than MR collisions, then the QPs the furthest away from the boundaries are less likely to undergo a dissipative collision. As a consequence, the QP flow becomes analogous to that of a viscous fluid in a channel (dubbed the Poiseuille flow). Such viscous corrections to electronic transport properties have been seen by a number of experiments^5–10^. All these studies were performed on mesoscopic ultra-pure metals. The strongest hydrodynamic signatures have been seen in graphene near the neutrality point and when electron velocity is set by the thermal energy. The velocity of degenerate electrons, on the other hand, is narrowly distributed around the Fermi velocity. Moreover, since the rate of electron-electron collisions is proportional to the square of the ratio of temperature to the Fermi temperature, MR collisions rarefy with increasing degeneracy.

Nevertheless, quantum liquids (such as both isotopes of helium) present hydrodynamic features associated with viscosity below their degeneracy temperature. Soon after the conception of Landau’s Fermi liquid theory, Abrikosov and Khalatnikov^11^ calculated the transport coefficients of an isotropic Fermi liquid, focusing on liquid ^3^He well below its degeneracy temperature. They showed that since the phase space for fermion-fermion scattering grows quadratically with temperature *T*, viscosity *η* (which is the diffusion constant for momentum) and thermal diffusivity *D* (which is the diffusion constant for energy) both follow *T*^−2^ and, as result, *κ* ∝ *T*^−1^. Subsequent theoretical studies^12,13^ confirmed this pioneering study and corrected^13^ the prefactors. Thermal conductivity^14,15^ and viscosity^16,17^ measurements at very low temperatures found the theoretically predicted temperature dependence for both quantities below *T* = 0.1K, deep inside the degenerate regime.

**Fig. 1:**
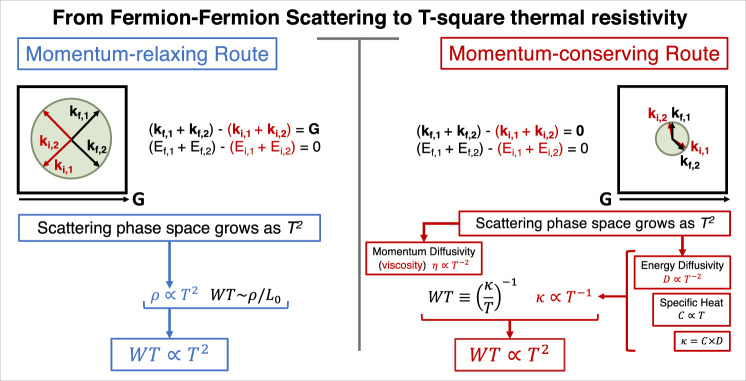
Two routes towards *T*^2^ thermal resistivity. *T*-square thermal resistivity in a Fermi liquid can arise in two distinct pictures of transport. The momentum-relaxing picture (left) is the one commonly used in metals. Because of the presence of a lattice, Umklapp collisions among electrons can occur. **k**_*i*,*j*_ and *E*_*i*,*j*_ respectively refer to the initial momentum and energy of electron *j* while **k**_*f*,*j*_ and *E*_*f*,*j*_ correspond to its final momentum and energy. These collisions decay the momentum current because a unit vector of the reciprocal lattice **G** is lost during the collision. The momentum-conserving picture (right) has been applied to the fermionic quasiparticles in ^3^He. We argue that it becomes relevant to metals, provided that : (i) Umklapp collisions become rare or impossible (because of the smallness of the Fermi radius) and (ii) the mean free path approaches the sample size.

However, the common picture of transport in metallic solids does not invoke viscosity (Fig. 1). The phase space for collisions among electronic quasiparticles is also proportional to the square of temperature. But the presence of a crystal lattice alters the context. Electron-electron collisions can degrade the flow of charge and heat by transferring momentum to the underlying crystal, if there is a finite amount of disorder. We will see below that if the electronic mean free path is sufficiently long compared to the sample dimensions, and if a significant portion of collisions conserve momentum (by avoiding Umklapp processes), then a finite *κT*∣_0_, equivalent to quadratic thermal resistivity ($WT={(\frac{\kappa }{T})}^{-1}$), caused by momentum-conserving collisions and evolving hand-in-hand with viscosity becomes relevant.

A fundamental correlation between the electronic thermal conductivity *κ*_*e*_ and the electrical conductivity *σ* is given by the Wiedemann–Franz (WF) law:

1 $$\frac{{\kappa }_{e}}{\sigma T}=\frac{{\pi }^{2}}{3}\frac{{k}_{B}^{2}}{{e}^{2}}$$

The left hand of the equation is the (electronic) Lorenz number, *L*_*e*_, which can be measured experimentally. The right-hand side is a fundamental constant, called the Sommerfeld value *L*_0_ = 2.44 × 10^−8^ V^2^ K^−2^. The WF law is expected to be valid when inelastic scattering is absent, i.e., at zero temperature.

Principi and Vignale (PV)^3^ recently argued that in hydrodynamic electron liquids, the WF law is violated because MC electron–electron (*e*–*e*) scattering would degrade thermal current but not electrical current. As a consequence, by drastically reducing the *L*_*e*_/*L*_0_ ratio, electron hydrodynamics would lead to a finite-temperature departure from the WF law. However, the standard transport picture based on MR collisions expects a similar departure at finite temperature as a consequence of inelastic small-angle *e*–*e* scattering^18–22^. The two pictures differ in an important feature: the evolution of the *L*_*e*_/*L*_0_ ratio with the carrier lifetime. In the hydrodynamic picture, the deviation from the WF law becomes more pronounced with the relative abundance of MC *e*–*e* collisions, which can be amplified by reducing the weight of MR collisions (by enhancing purity or size).

Here, we present a study of heat and charge transport in semi-metallic antimony (Sb) and find that *κ* and *σ* both increase with sample size. Sb is the most magnetoresistant semi-metal^23^. The mean-free-path *ℓ*_0_ of its extremely mobile charge carriers depends on the thickness of the sample at low temperature^24^. We begin by verifying the validity of the WF law in the zero-temperature limit and resolving a clear departure from it at finite temperature. This arises because of the inequality between the prefactors of the *T*-square electrical and thermal resistivities^21^. In contrast to its electrical counterpart, the *T*-square thermal resistivity (which is equivalent to *κ* ∝ *T*^−1^), can be purely generated by MC scattering which sets the viscosity of the electronic liquid. We find that the departure from the WF law is amplified with the increase in the sample size and the carrier mean free path, in agreement with the hydrodynamic scenario^3^. We then quantify *κT*∣_0_ and the quadratic lifetime of fermion-fermion collisions, *τ*_*κ*_*T*^2^, for electrons in Sb and compare it with that of ^3^He fermions.

## Results

### The band structure

**Fig. 2:**
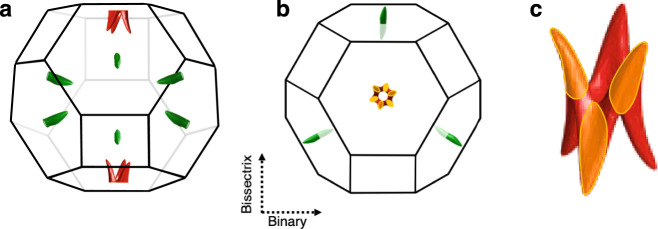
Fermi surface and the Brillouin zone of antimony (Sb). **a** The Fermi surface consists of electron pockets (in green) and hole pockets (in red). All pockets are located at zone boundaries and have a mirror counterpart due to the inversion symmetry. The Brillouin zone of the A7 crystal structure, nearly a truncated cuboctahedron, is shown by black solid lines. **b** Projection to the trigonal plane. **c** The Fermi surface of holes centered at the T-point. This structure, dubbed ZONY^23^, consists of six interconnected pockets (shown in orange).

  Figure 2 shows the Fermi surface and the Brillouin Zone (BZ) of antimony^23,25–28^. In this compensated semi-metal, electron pockets are quasi-ellipsoids located at the L-points of the BZ. The valence band crosses the Fermi level near the T-points of the Brillouin zone generating a multitude of hole pockets. The tight-binding picture conceived by Liu and Allen^28^, which gives a satisfactory account of experimental data, implies that these pockets are not six independent ellipsoids scattered around the T-point^26^, but a single entity^23^ centered at the T-point formed by their interconnection (see Fig. 2c).

One important point is that the pockets are small. The largest Fermi wave-vector is 0.22 times the reciprocal lattice parameter^23,28^. Since in an Umklapp collision between electrons, the sum of the Fermi wave-vectors should exceed the width of the BZ, Umklapp events cannot occur when *k*_*F*_ < 0.25. The fact that the FS pockets are too small to allow Umklapp events will play an important role below.

### Electrical and thermal transport measurements

**Fig. 3:**
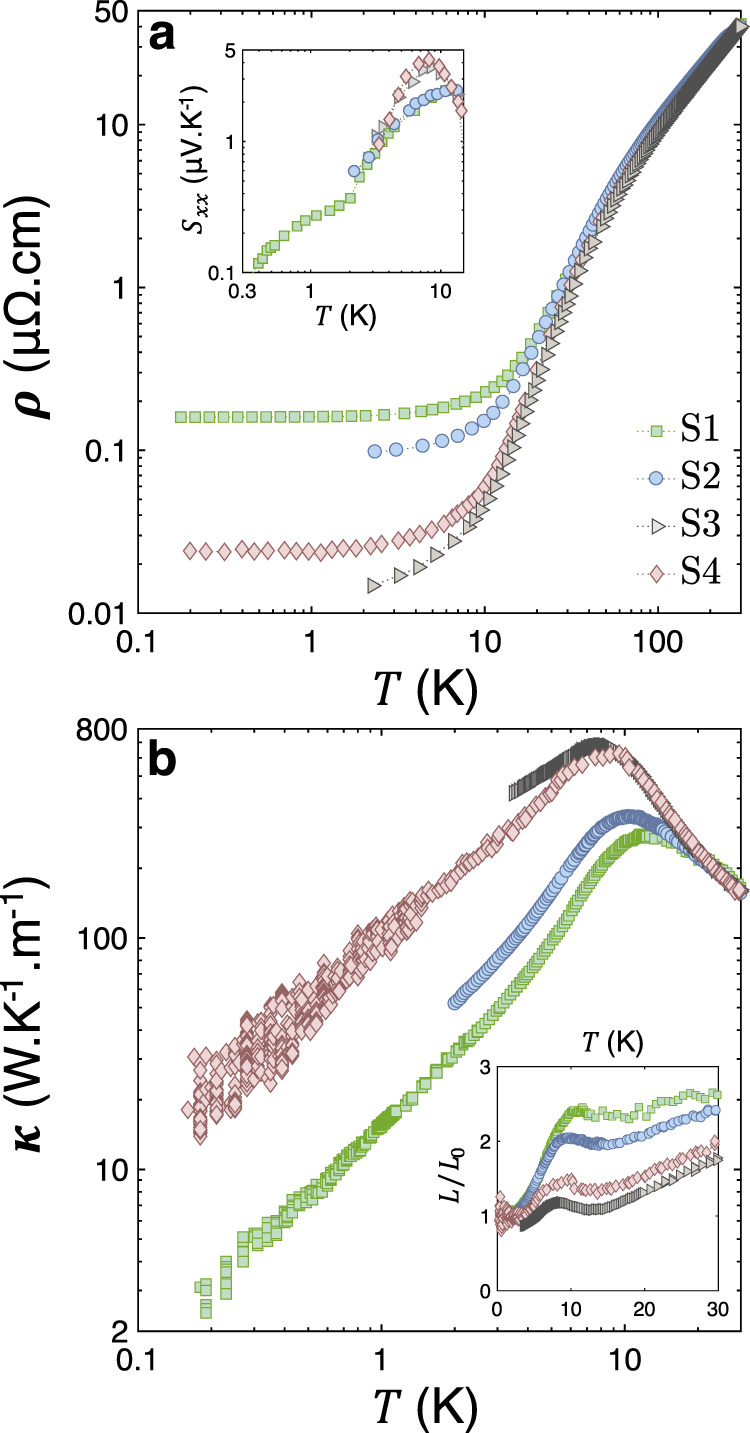
Zero-field transport properties. **a** Electrical resistivity along the bisectrix direction, *ρ*, plotted as a function of temperature for the various sizes of Sb samples presented in Table 1. Inset shows the zero-field thermopower *S*_*xx*_ as a function of the temperature of the same samples. **b** Temperature dependence of the thermal conductivity, *κ*, of the aforementioned Sb samples. Inset shows the Lorenz number *L* plotted as *L*/*L*_0_, where *L*_0_ is the Sommerfeld number, as a function of temperature. *L*/*L*_0_ = 1 corresponds to the recovery of the Wiedemann–Franz law.

All measurements were carried out using a conventional 4-electrode (two thermometers, one heater, and a heat sink) setup (further details are given in the Method section). The Sb crystals are presented in Table 1. Electrical and heat currents were applied along the bisectrix direction of all samples. The electrical resistivity, shown in Fig. 3a, displays a strong size dependence below *T* = 25K and saturates to larger values in the two thinner samples, as reported previously^24^. As seen in Table 1, the mean free path remains below the average thickness, but tends to increase with the sample average thickness.

**Table 1:** Details of the samples.

Sample	Size (mm^3^)	RRR	*ρ*_0_ (n*Ω* cm)	$\overline{{\rm{s}}}$ (*μ*m)	*ℓ*_0_ (*μ*m)	*ρ*_0_$\overline{{\rm{s}}}$ (p*Ω* m^2^)	A_2_ (n*Ω* cm K^−2^)	B_2_ (n*Ω* cm K^−2^)
1	([0.25 ± 0.05 × 0.5 × 4.1)	260	159	350	17	0.56	0.70 ± 0.03	0.81 ± 0.05
1b	(0.2 × 0.5 × 4.6)	250	164	320	16	0.49	0.73 ± 0.04	–
2	(0.4 × 0.4 × 4.1)	430	94.6	400	28	0.38	0.56 ± 0.03	0.74 ± 0.03
3	(1.1 × 1.0 × 10.0)	3000	13.4	1050	197	0.14	0.38 ± 0.03	0.68 ± 0.04
3^*^	(1.1 × 1.0 × 7.0) (cut from 3)	3000	13.4	1050	197	0.14	0.38 ± 0.03	–
4	(1.0 × 5.0 × 10.0)	1700	24.1	2240	110	0.54	0.32 ± 0.04	0.63 ± 0.08
5	(3.0 × 1.0 × 10.0)	3700	11.1	1730	238	0.19	0.33 ± 0.03	–
6	(1.7 × 1.8 × 10.0)	4200	9.8	1800	270	0.18	0.33 ± 0.03	–

Sb crystals used in this study were oriented along the bisectrix crystallographic axis. $\overline{s}=\sqrt{{\rm{width}}\times {\rm{thickness}}}$ represents the average diameter of the conducting cross-section. The residual resistivity ratio is defined as $RRR=\frac{{\rho }_{300K}}{{\rho }_{0}}$. The carrier mean free path *ℓ*_0_ was calculated from the residual resistivity and the expression for Drude conductivity assuming three spherical hole and three spherical electron pockets. This is a crude and conservative estimation, because the mean free path of hole-like and electron-like carriers residing in different valleys is likely to differ (See the Supplementary Note 2 for more details). Also given is the product of ${\rho }_{0}\overline{s}$, a measure of crystalline perfection (Supplementary Note 2). The last two columns give the electrical (*A*_2_) and thermal (*B*_2_) *T*^2^-resistivities prefactors.

The thermal conductivity, *κ*, of the same samples is presented in Fig. 3b. *κ* presents a peak whose magnitude and position correlates with sample size and resistivity. In large samples the peak is larger in amplitude and occurs at lower temperatures. Semi-metallic antimony has one electron and one hole for  ~600 atoms. The lattice and electronic contributions to the thermal conductivity are comparable in size. The inset of Fig. 3a shows the temperature dependence of the Seebeck coefficient in the same samples. The Seebeck coefficient remains below 5 *μ*V/K, as reported previously^26^, because of the cancellation between hole and electron contributions to the total Seebeck effect. The small size of the Seebeck response has two important consequences. First, it implies that the thermal conductivity measured in absence of charge current is virtually identical to the one measured in absence of electric field (which is the third Onsager coefficient^29^). The second is that the ambipolar contribution to the thermal transport is negligible and *κ* = *κ*_*e*_ + *κ*_*ph*_ (see the Supplementary Notes 5 and 6 respectively for a discussion of both issues).

The temperature dependence of the overall Lorenz number (*L* = (*κρ*/*T*)) divided by *L*_0_, is plotted as a function of temperature in the inset of Fig. 3b. For *T* < 4K, *L*/*L*_0_ → 1. The Wiedemann-Franz law is almost recovered below *T* = 4K in all samples. At higher temperatures, *L* displays a non-monotonic and size-dependent temperature dependence resulting from two different effects: a downward departure from the WF law in *κ*_*e*_ and a larger share of *κ*_*ph*_ in the overall *κ*.

**Fig. 4:**
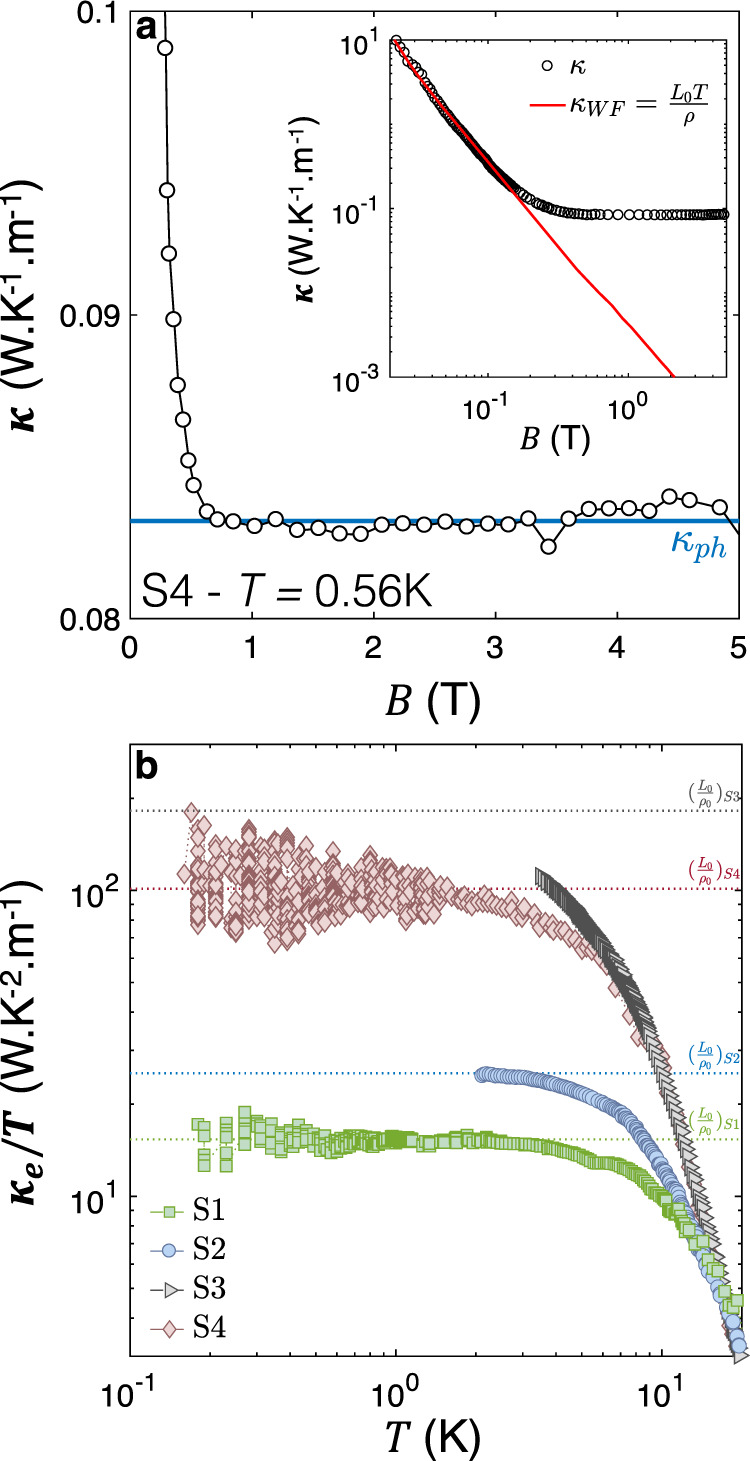
Using a magnetic field to extract electronic and phononic components of thermal conductivity. **a** Magnetic field dependence of the thermal conductivity of sample S4 at *T* = 0.56K. The averaged field-independent fraction of *κ*, associated with the phonon contribution to *κ* is shown as *κ*_*ph*_. The inset shows a comparison of *κ* and ${\kappa }_{WF}=\frac{T{L}_{0}}{\rho (B)}$ as a function of the magnetic field. For *B* > 0.5T, the electronic thermal conductivity becomes negligible in regard of the phonon contribution. **b** Temperature dependence of the electronic thermal conductivity *κ*_*e*_ = *κ* − *κ*_*ph*_ plotted as *κ*_*e*_/*T*. Horizontal lines representing *L*_0_/*ρ*_0_ for the various samples are featured in the graph.

**Fig. 5:**
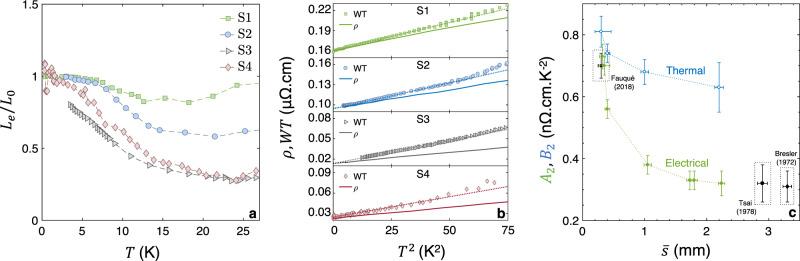
The WF law, the *T*-square resistivities, and their evolution with disorder. **a** Electronic fraction of the Lorenz number *L*_*e*_ = *κ*_*e*_*ρ*/*T* plotted as *L*_*e*_/*L*_0_, where *L*_0_ is the Sommerfeld number, as a function of temperature. *L*_*e*_/*L*_0_ = 1 corresponds to the recovery of the Wiedemann-Franz law. **b** Thermal (*WT*) and electrical (*ρ*) resistivities plotted as functions of *T*^2^ for the four sizes of Sb samples. *WT* is featured as symbols while *ρ* is shown as a solid line. All four graphs share a common x-axis and y-axis span. **c** Evolution of the electrical and thermal *T*^2^-resistivities prefactors with sample size in Sb. Data points from^23,32,33^ are featured. Error-bars along the x-axis are defined by the uncertainty on the geometry of the samples while they are defined along the y-axis by the standard deviation of the *T*^2^-fit to the resistivity data.

The application of a magnetic field provides a straightforward way to separate *κ*_*e*_ and *κ*_*ph*_ in a semi-metal with very mobile carriers^30^. Indeed, under the effect of a magnetic field, the electronic conductivity drastically collapses (the low-temperature magnetoresistance in Sb reaches up to 5.10^6^% at *B* = 1T, as shown in Supplementary Fig. 1) while the lattice contribution is left virtually unchanged. This is visible in the field dependence of *κ*, shown in Fig. 4a (for sample S4 at *T* = 0.56K). One can see a sharp drop in *κ*(*B*) below B^*^ ≈ 0.5T and a saturation at higher fields. The initial drop represents the evaporation of *κ*_*e*_ due to the huge magnetoresistance of the system. The saturation represents the indifference of *κ*_*ph*_ towards the magnetic field. This interpretation is confirmed by the logarithmic plot in the inset and is further proven by the study of the low-temperature thermal conductivity of Sb as a function of temperature under the effect of several fields presented in Supplementary Fig. 4. Below B^*^ ≈ 0.1T, *L*_0_*T*/*ρ* is close to *κ*, indicating that in this field window, heat is carried mostly by electrons and the WF law is satisfied. However, by B^*^ ≈ 1T, *L*_0_*T*/*ρ* is three orders of magnitude lower than *κ*, implying that at this field, heat is mostly carried by phonons with a vanishing contribution from electrons. The electronic component of thermal conductivity separated from the total thermal conductivity, (*κ*_*e*_(*T*) = *κ*(*B* = 0)(*T*) − *κ*(*B* = 1*T*)(*T*)) is shown in Fig. 4b. One can see that, for all four samples and at sufficiently low temperature, *κ*_*e*_/*T* becomes constant (and equal to *L*_0_/*ρ*_0_). It is the subsequent downward deviation at higher temperatures that will become the focus of our attention. We construct the electronic Lorenz ratio *L*_*e*_ = *κ*_*e*_*ρ*/*T* and show its evolution with temperature in Fig. 5a. Below *T* < 4K, *L*_*e*_ ≃ *L*_0_ in all samples, save for S3, the cleanest. With increasing temperature, *L*_*e*_/*L*_0_ dives down and the deviation becomes larger as the samples become cleaner.

Let us scrutinize separately the temperature dependence of the electrical and the thermal resistivities. The latter can be expressed in the familiar units of resistivity (i.e., *Ω*m), using *WT* = *L*_0_*T*/*κ*_*e*_ as a shorthand. Figure 5b shows *ρ* and *WT* as a function of *T*^2^ for the four different samples. In the low-temperature limit, an asymptotic *T*^2^ behavior is visible in all samples and the two lines corresponding to *ρ* and *WT* have identical y-axis intercepts, thus confirming the recovery of the WF Law in the zero-temperature limit. In every case, the slope of *WT*(*T*^2^) is larger than that of *ρ*(*T*^2^), indicating that the prefactor of the thermal-*T*-square resistivity (dubbed *B*_2_) is larger than the prefactor of the electrical-*T*-square resistivity (dubbed *A*_2_). This behavior, observed for the first time in Sb, was previously reported in a handful of metals, namely W^19^, WP_2_^21^, UPt_3_^31^, and CeRhIn_5_^20^.

## Discussion

*T*-square resistivity arises due to *e*–*e* collisions. In the momentum-relaxing picture, the common explanation for the experimentally observed *B*_2_ > *A*_2_ inequality is the under-representation of small-angle scattering in the electrical channel, which damps the electric prefactor *A*_2_, but not its thermal counterpart *B*_2_^18–22^. This picture cannot explain that, as seen in Fig. 5b, the two slopes are further apart in the cleaner samples. The evolution of the two prefactors with sample dimensions is presented in Fig. 5c. The figure also includes previous data on the slope of electrical *T*^2^-resistivity^23,32,33^. One can see the emergence of a consistent picture: the electrical (*A*_2_) prefactor displays a significant size dependence and the *A*_2_/*B*_2_ ratio substantially decreases with the increase in sample size and electronic mean free path.

Because of momentum conservation, *e*−*e* collisions cannot decay the momentum flow by themselves. Such collisions can relax momentum through two mechanisms known as Umklapp and interband (or Baber) scattering. There are two known cases of *T*-square resistivity in absence of either mechanisms^34,35^.

The smallness of the Fermi surface in Sb excludes the Umklapp mechanism. However, the interband mechanism is not excluded. It can generate both a *T*-square and a *A*_2_/*B*_2_ ratio lower than unity^22^. Li and Maslov^22^ have argued that the ratio of the two prefactors (and therefore the deviation from the WF law) in a compensated semi-metal like Sb is tuned by two material-dependent parameters: (i) the screening length and (ii) the relative weight of interband and intraband scattering. In their picture, increasing the screening length would enhance *B*_2_ and leave *A*_2_ unchanged. Enhancing interband scattering would also reduce the Lorenz ratio. Given that neither of these two is expected to change with the crystal size or imperfection, the evolution seen in Fig. 5c cannot be explained along either of these two lines.

**Fig. 6:**
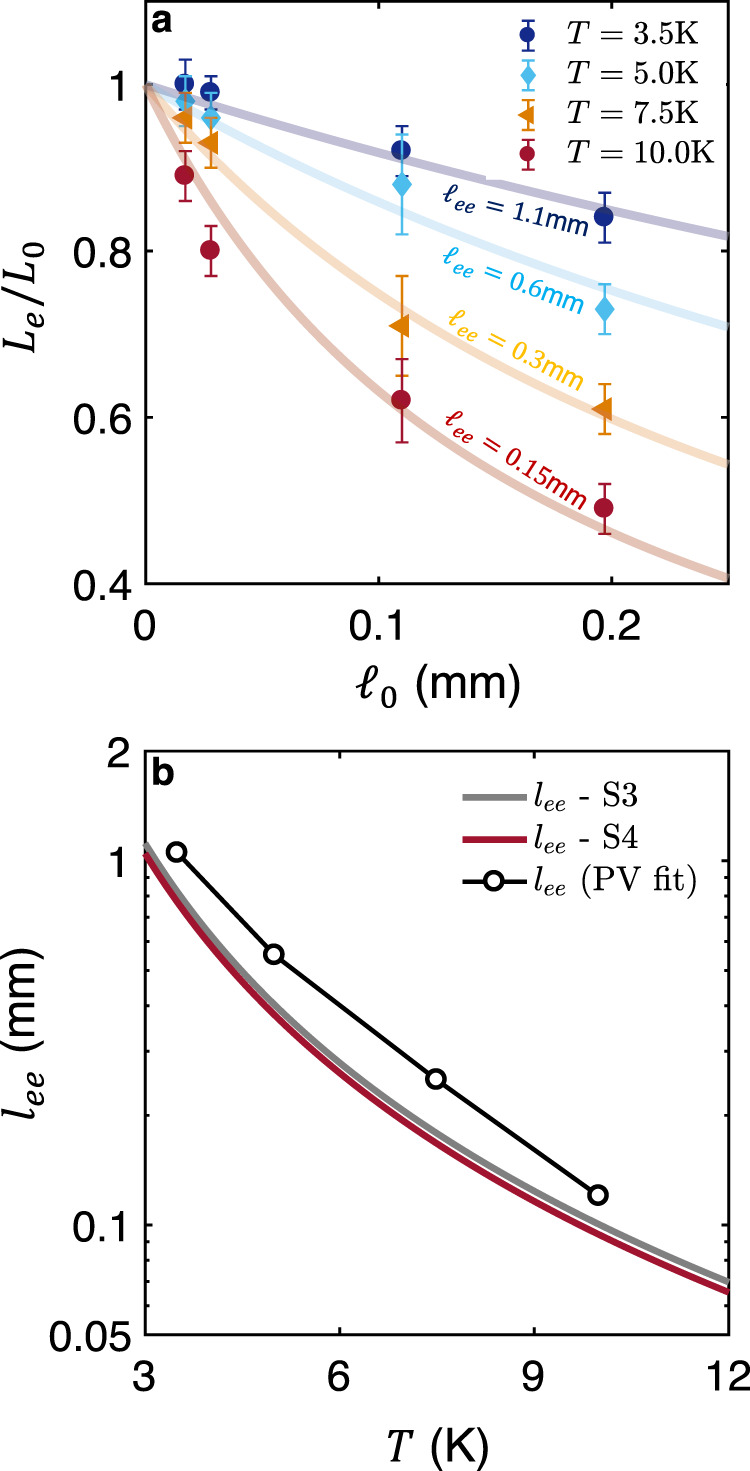
The evolution of the Wiedemann-Franz correlation with the ratio of momentum-relaxing and momentum conserving mean free paths. **a** The electronic Lorenz number *L*_*e*_ at *T* = 10K, normalized by the Sommerfeld value *L*_0_, plotted as a function of the residual mean free path *ℓ*_0_ at various temperatures. The solid lines correspond to a fit given by the equation $L/{L}_{0}=1/({1+{\ell }_{0}/{\ell }_{ee}})$ proposed by Principi and Vignale (PV) ^3^. *ℓ*_0_ refers to the zero-temperature Drude mean free path while *ℓ*_*ee*_(*T*) is the typical distance traveled by a charge carrier in-between two momentum-conserving collisions. Error bars are defined from the experimental uncertainty on *L*_*e*_ featured in Fig. 5a. **b** Comparison of *l*_*ee*_ determined by the fit to the aforementioned PV formula and what is yielded by assuming that the difference between the two *T*-square resistivities represents the fraction of collisions which conserve momentum. In that case, ${\ell }_{ee}=\frac{{\ell }_{0}{\rho }_{0}}{({B}_{2}-{A}_{2}){T}^{2}}$.

In contrast, the hydrodynamic picture provides a straightforward account of our observation. The Principi and Vignale scenario^3^ predicts that the deviation from the WF law should become more pronounced with increasing carrier lifetime (or equivalently mean free path *ℓ*_0_): *L*_*e*_/*L*_0_ = 1/(1 + *ℓ*_0_/*ℓ*_*ee*_). Such a picture provides a reasonable account of our observation, as seen in Fig. 6a, which shows the variation of *L*_*e*_/*L*_0_ at different temperatures with carrier mean free path. In this picture, the evolution of the Lorenz ratio with *ℓ*_0_ would imply a mean free path for MC *e*−*e* scattering, *ℓ*_*ee*_, which ranges from 0.15 mm at *T* = 10K to 1.1 mm at *T* = 3.5K.

These numbers are to be compared with *ℓ*_*ee*_ extracted from the magnitude of (*B*_2_,*A*_2_), assuming that MC *e* − *e* collisions generate the difference between these two quantities and the Drude formula. As seen in Fig. 6b, while the two numbers closely track each other between *T* = 3K and *T* = 10K, a difference is found. *ℓ*_*ee*_ extracted from the isotropic Drude formula is 1.6 times smaller than the one yielded by the isotropic Principi-Vignale formula. Now, the electronic structure of antimony is strongly anisotropic with a tenfold difference between the longest and the shortest Fermi wave-vectors along different orientations^28^. In such a context, one expects an anisotropic *ℓ*_*ee*_, with different values along different orientations. Moreover, intervalley scattering between carriers remaining each in their only valley and scattering between electrons and holes should also have characteristic length scales. Therefore, the present discrepancy is not surprising and indicates that at this stage, only the order of magnitude of the experimental observation is accounted for by a theory conceived for isotropic systems^3^. Note the macroscopic ( ~ mm) magnitude of *ℓ*_*ee*_ near *T* ~ 4K which reflects the fact that electrons are in the ultra-degenerate regime (*T*/*T*_*F*_ ~ 4 × 10^−3^) and therefore, the distance they travel to exchange momentum with another electron is almost six orders of magnitude longer than the distance between two electrons.

An account of boundary scattering is also missing. The decrease in *ρ*_0_ with sample size in elemental metals have been widely documented and analyzed by pondering the relative weight of specular and diffusive scattering^36^. This can also weigh on the magnitude of *A*_2_^37^. However, a quantitative account of the experimental data, by employing Soffer’s theory^38^, remains unsuccessful^24,37,39^. The role of surface roughness acquires original features in the hydrodynamic regime^40^, which are yet to be explored by experiments on samples with mirror/matt surface dichotomy.

Having shown that the experimentally-resolved *T*^2^ resistivity is (at least) partially caused by thermal amplification of momentum exchange between fermionic quasiparticles, we are in a position to quantify *κT*∣_0_ in antimony and compare it with the case of ^3^He.

Its lower boundary is *L*_0_/(*A*_2_−*B*_2_) and the upper boundary *L*_0_/*B*_2_. This yields 3900 < *κT*∣_0_ < 7900 in units of W m^−1^. This is six orders of magnitude larger than in normal liquid ^3^He^15^ (see Table 2). Such a difference is not surprising since: (i) *κT*∣_0_ of a Fermi liquid is expected to scale with the cube of the Fermi momentum (*p*_*F*_) and the square of the Fermi velocity (*v*_*F*_)^41^; and (ii) ^3^He is a strongly correlated Fermi liquid while Sb is not. More specifically *κT*∣_0_ can be written in terms of the Fermi wave-vector (*k*_*F*_) and the Fermi energy (*E*_*F*_):

2 $$\kappa T{| }_{0}=\frac{1}{{B}_{0}}\frac{{E}_{F}^{2}{k}_{F}}{\hslash }$$

**Table 2:** Comparison of two Fermi liquids.

System	Density (cm^−3^)	T_*F*_ (K)	k_*F*_ (nm^−1^)	*κT*∣_0_ (W m^−1^)	$\frac{{{\rm{E}}}_{{\rm{F}}}^{2}{{\rm{k}}}_{{\rm{F}}}}{\hslash }$ (W m^−1^)	B_0_	*τ*_*κ*_T^2^(s K^2^)
^3^He^15^	1.63 × 10^22^	1.8	7.8	2.9 × 10^−4^	0.04	137	3.9 × 10^−13^
Sb	*n* + *p* = 1.1 × 10^20^	1100^28^	0.8 (average)^28^	3900–7900	1500	0.4–0.8	1.5-3.0 × 10^−8^

The density of atoms at ambient pressure in ^3^He is two orders of magnitude larger than the total density of electron-like and hole-like carriers (*n* + *p*) in Sb. Also listed in this Table are the average Fermi temperature, the average Fermi momentum and the magnitude of the experimentally-resolved *κT*∣_0_ (W.m^−1^). Its natural units are $\frac{{E}_{F}^{2}{k}_{F}}{\hslash }$. *B*_0_ is defined in the main text. *τ*_*k*_*T*^2^ quantifies the rate of fermion-fermion collisions.

This equation is identical to equation 17 in ref. ^41^. The dimensionless parameter *B*_0_ (See Supplementary Note 7 for more details) quantifies the cross-section of fermion-fermion collisions.

In the case of ^3^He, measuring the temperature dependence of viscosity^16,17^ leads to $\eta {T}_{0}^{2}$ and measuring the temperature dependence of thermal conductivity^15^ leads to *κT*∣_0_. The rate of fermion-fermion collisions obtained with these two distinct experimental techniques are almost identical : *τ*_*η*_*T*^2^ ≈ *τ*_*κ*_*T*^2^^17^. Calkoen and van Weert^41^ have shown that the agreement between the magnitude of *κT*∣_0_, the Landau parameters and the specific heat^42^ is of the order of percent.

^3^He is a dense strongly-interacting quantum fluid, which can be solidified upon a one-third enhancement in density. As a consequence, *B*_0_ ≫ 1. In contrast, the electronic fluid in antimony is a dilute gas of weakly interacting fermions and *B*_0_ is two orders of magnitude lower, as one can see in Table 2. The large difference in *B*_0_ reflects the difference in collision cross-section caused by the difference in density of the two fluids.

The *T*^2^ fermion-fermion scattering rate can be extracted and *τ*_*κ*_*T*^2^ can be compared with the case of ^3^He^15–17,43^ (See Table 2). As expected, it is many orders of magnitude smaller in Sb than in its much denser counterpart. A similar quantification is yet to be done in strongly-correlated electronic fluids.

In summary, we found that the ratio of the thermal-to-electrical *T*-square resistivity evolves steadily with the elastic mean free path of carriers in bulk antimony. The momentum-conserving transport picture provides a compelling explanation for this observation. In this approach, thermal resistivity is in the driver’s seat and generates a finite electrical resistivity which grows in size as the sample becomes dirtier.

This a hydrodynamic feature, since the same fermion–fermion collisions, which set momentum diffusivity (that is viscosity) set energy diffusivity (the ratio of thermal conductivity to specific heat). Note that this is a feature specific to quantum liquids, in contrast to the upward departure from the WF law reported in graphene when carriers are non-degenerate^7^.

The observation of this feature in Sb was made possible for a combination of properties. (i) The mean free path of carriers was long enough to approach the sample dimensions; (ii) The Normal collisions outweigh Umklapp collisions because the Fermi surface radii of all pockets are less than one-fourth of the width of the Brillouin zone. Finally, at the temperature of investigation, resistive scattering by phonons is marginal. All these conditions can be satisfied in low-density semimetals such as Bi^44^ or WP_2_^9,21^. In contrast, in a high-density metal such as PdCoO_2_^6^, such a feature is hard to detect. Not only, due to the large Fermi energy, the *T*-square resistivity is small and undetectable^45^, but also due to the large Fermi radius^46^, electron–electron collisions are expected to be mostly of Umklapp type.

Beyond weakly correlated semi-metals, our results point to a novel research horizon in the field of strongly correlated electrons. One needs quasi-ballistic single crystals (which can be provided thanks to Focused-Ion-Beam technique) of low-density correlated metals. URu_2_Si_2_^47^ and PrFe_4_P_12_^48^, known to be low-density strongly correlated Fermi liquids, appear as immediate candidates but other systems may qualify. The electron-electron collision cross-section, which can be quantified by a study similar to ours should be much larger than what is found here for a weakly correlated system such as Sb.

## Methods

### Samples

Sb crystals were commercially obtained through MaTeck GmbH. Their dimensions are given in Table 1 of the main text. Samples S1, S1b, and S2 were cut from a ingot of Sb using a wire saw. Samples S3, S4, S5, and S6 were prepared by MaTeck to the aforementioned dimensions: sample S4 was cut while samples S3, S5, and S6 were etched to these dimensions. Sample S3 was measured before and after a cut of a few mm perpendicular to the bisectrix direction. The long axis of all samples were oriented along the bisectrix direction.

### Measurements

The thermal conductivity measurements were performed with a home-built one-heater-two-thermometers set-up. Various thermometers (Cernox chips 1010 and 1030 as well as RuO_2_) were used in this study. Our setup was designed to allow the measurement of both the thermal conductivity, *κ* and the electrical resistivity, *ρ* with the same electrodes.

The thermometers were either directly glued to the samples with Dupont 4922N silver paste or contacts were made using 25 *μ*m-diameter silver wires connected to the samples via silver paste (Dupont 4922N). Contact resistance was inferior to 1*Ω*. The thermometers were thermally isolated from the sample holder by manganin wires with a thermal conductance several orders of magnitude lower than that of the Sb samples and silver wires. The samples were connected to a heat sink (made of copper) with Dupont 4922N silver paste on one side and to a RuO_2_ chip resistor serving as a heater on the other side. Both heat and electrical currents were applied along the bisectrix direction. The heat current resulted from an applied electrical current*I* from a DC current source (Keithley 6220) to the RuO_2_ heater. The heating power was determined by *I* × *V* where *V* is the electric voltage measured across the heater by a digital multimeter (Keithley 2000). The thermal conductivity was checked to be independent of the applied thermal gradient by changing *ΔT*/*T* in the range of 10%. Special attention was given not to exceed *ΔT*/*T*∣_*max*_  = 10%.

The thermometers were calibrated in-situ during each experiment and showed no evolution with thermal cycling. Special attention was given to suppress any remanent field applied to the sample and self-heating effects.

The accuracy of our home-built setup was checked by the recovery of the Wiedemann–Franz law in an Ag wire at *B* = 0T and *B* = 10T through measurements of the thermal conductivity and electrical resistivity. At both magnetic fields, the WF was recovered at low temperatures with an accuracy of 1%^21^.

## Supplementary information

Supplementary Information

## Data Availability

All data supporting the findings of this study are available from the corresponding authors upon request.
