# Supplementary material for: Thermal resistivity and hydrodynamics of the degenerate electron fluid in antimony
Source: Nat Commun. 2021 Jan 8;12:195. doi: 10.1038/s41467-020-20420-9 (PMC7794374; doi:10.1038/s41467-020-20420-9)
Supplement: Supplementary file 1 — Supplementary Information [file 41467_2020_20420_MOESM1_ESM.pdf]

Supplementary Material for ’Thermal resistivity and hydrodynamics of the degenerate electron fluid in antimony’

I. SUPPLEMENTARY FIGURES

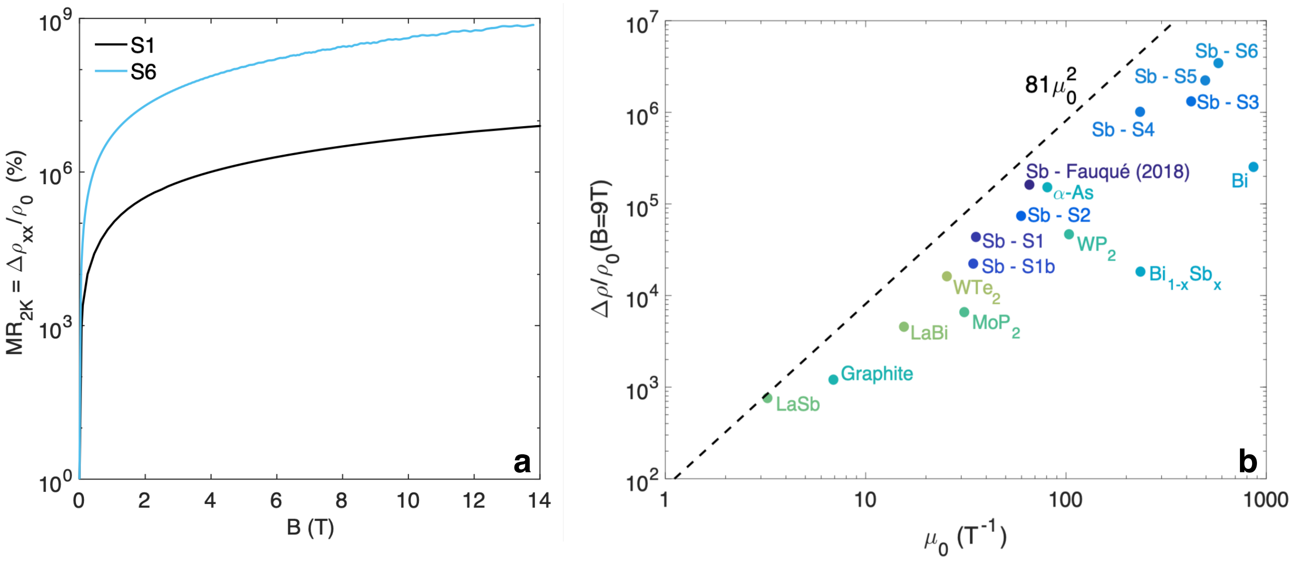

Supplementary Figure 1 : **Magnetoresistance of Sb. a** Magnetoresistances of sample S1 and S6 at $T = 2$K. **b** Magnetoresistance of various semi-metals at $B = 9$T and $T = 2$K as a function of the mobility $\mu_0 = 1/(\rho_0(n+p)e)$ where e is the elementary charge, n and p are the electron and hole densities and ρ_0 the zero field resistivity at $T = 2$K. μ_0 is expressed in Tesla^{-1} $= 10^4$cm^2.V^{-1}.s^{-1}

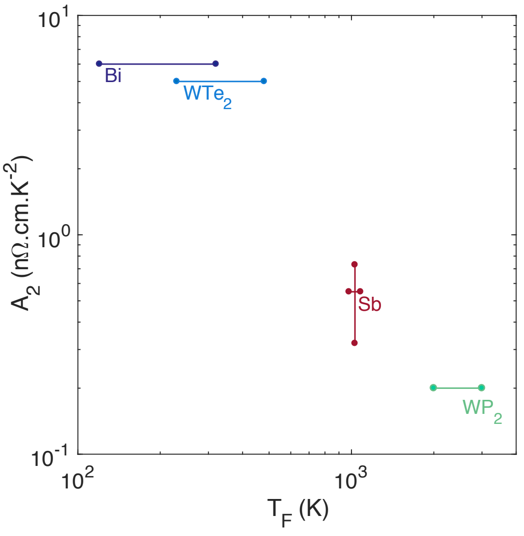

Supplementary Figure 2 : **Amplitude of the T^2-resistivity prefactor in semi-metals. a** Electrical T^2-resistivity prefactor (A_2) plotted as a function of the Fermi temperature for the semi-metals discussed in table 1. T_F was taken for both electrons and holes for Sb [1, 2], Bi [2, 3], WTe_2 [4] and WP_2 [5, 6] and the prefactor A_2 for Sb was taken from this work, Bi [1], WTe_2 [4] and WP_2 [7].

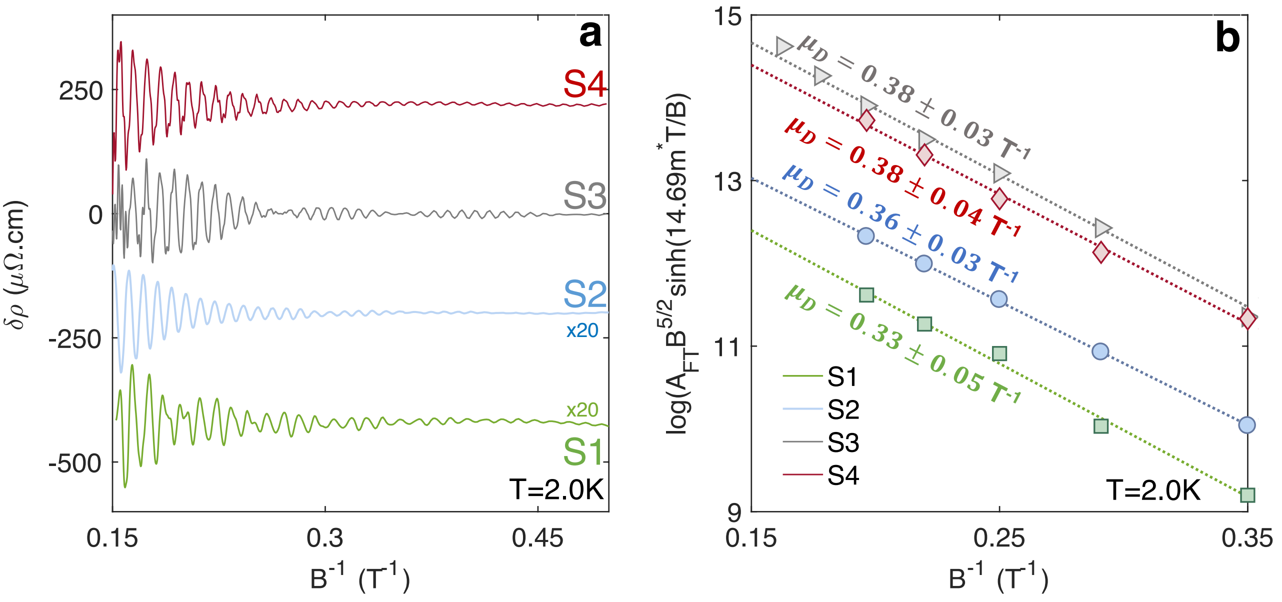

Supplementary Figure 3 : **Effect of sample size on the quantum oscillations observed in Sb.** **a** Quantum oscillations of the magnetoresistance (the Shubnikov-de Haas effect) in four Sb samples as listed in table 1 of the main text. In all cases, the field was applied along the trigonal axis and the current was applied along the bisectrix axis. **b** Dingle analysis of the data revealing a quasi-identical mobility.

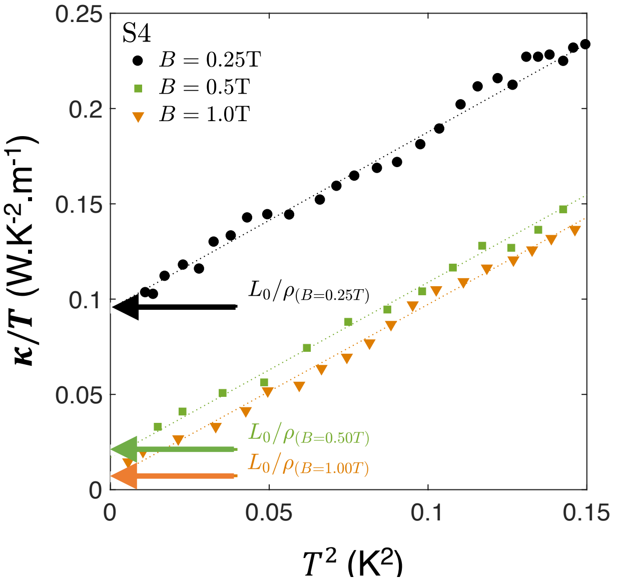

Supplementary Figure 4 : **Recovery of Wiedemann-Franz law at small applied magnetic fields.** **a** Thermal conductivity plotted as κ/T as a function of T^2 in sample S4 for three successive magnetic fields. The symbols show the experimental values of κ/T and the dotted lines are the linear fit while the value of L_0/ρ_B are featured as arrows. The recovery of the Wiedemann-Franz law at each magnetic field is shown by the intercept of the dotted lines and arrows.

II. SUPPLEMENTARY TABLES

Semi-metal	$n = p$ (cm^{-3})	$\overline{\rho_0}$ ($\mu\Omega$.cm)	$\overline{\mu_0}$ ($\mathrm{m}^2.\mathrm{V}^{-1}.\mathrm{s}^{-1}$)	m^* (m_0)	$\overline{T_{F,e}}$ (K)	$\overline{T_{F,h}}$ (K)	References
Sb	5.5×10^{19}	~ 0.05	~ 500	$0.07 - 1$	1080	980	[1, 2]
Bi	3.0×10^{17}	~ 1	~ 1000	$0.001 - 0.612$	320	120	[2, 3]
WP_2	2.5×10^{21}	~ 0.005	~ 400	$0.7 - 1.9$	3000	2000	[5, 6]
WTe_2	6.8×10^{19}	~ 1	~ 5	$0.1 - 1.2$	480	230	[4]

Supplementary Table 1 : **Comparison of prominent semi-metals.** We compare the electronic concentration n, typical residual resistivity $\overline{\rho_0}$, typical electronic mobility $\overline{\mu_0}$, carriers effective mass m^* and typical Fermi temperature of electrons $\overline{T_{F,e}}$ and holes $\overline{T_{F,h}}$ in these materials. References used to construct this table are featured in the last column.

Sample	ρ_0(nΩ cm)	μ_0 ($\mathrm{m}^2\mathrm{V}^{-1}\mathrm{s}^{-1}$)	μ_D ($\mathrm{m}^2\mathrm{V}^{-1}\mathrm{s}^{-1}$)	r
1	159	71	0.33	215
2	94.6	120	0.36	333
3	13.4	848	0.38	2231
4	24.1	772	0.38	2031

Supplementary Table 2 : **The two mobilities in four different crystals**. Transport mobility, μ_0 has been extracted from ρ_0 using $\mu_0 = 1/\rho_0 e(n+p)$ and Dingle mobility μ_D is extracted from a Dingle analysis of the quantum oscillations. r is the ratio of the two mobilities.

III. SUPPLEMENTARY NOTES

A. Supplementary Note 1 : Magnetoresistance and mobility.

The high mobility of charge carriers in Sb leads to a very large magnetoresistance, as reported in Ref.[8]. The samples presented in this study confirm this. As an example, the magnetoresistance of sample S6 at $T = 2$K and $B = 9$T is shown in Supplementary Figure 1.a. This large magnetoresistance translates into a suppression of the electronic thermal conductivity through the Wiedemann-Franz law. As a consequence, the separation of lattice and electronic contributions of κ becomes straightforward. The mobility and the magnetoresistance of the samples used in this study are shown in Supplementary Figure 1.b and compared to other semi-metals. One can see that carriers in Sb are extremely mobile compared to most other semi-metals.
Supplementary Table 1 compares the electronic properties of Sb with a few other semi-metals. Supplementary Figure 2 shows the magnitude of the electrical T^2-resistivity prefactor A_2 in four different semi-metals. One can see that A_2 decreases with increasing Fermi temperature, as previously noted in the case of numerous dilute metals [9]. The correlation between A_2 and E_F^2 is an extension of the Kadowaki-Woods correlation [10] to low-density systems [11].

B. Supplementary Note 2 : Estimation of the electronic mean-free-path

In the Drude picture, the measured residual resistivity, ρ_0 is related to the scattering time of electrons and holes and their masses by Supplementary Eq.(1):

$$\rho_0^{-1} = e^2 \left(\frac{n\tau_e}{m_e^*} + \frac{p\tau_h}{m_h^*} \right) \tag{1}$$

In Sb, the compensation between electron and hole densities holds with an accuracy of 10^{-4} and one has: $n = p = 5.5 \times 10^{19}$ cm^{-3} [8]. However, electrons and hole pockets have different shapes, significant mass anisotropy and are not aligned parallel to each other. Their associated scattering time is unlikely to be identical.

The mean-free-path of the samples given in table 1 of the main text was extracted from their residual resistivity using a conservative and crude approximation. If the Fermi surface is composed of z_h spheres for hole-like and z_e spheres for electron-like carriers, then the average Fermi wave-vector for both $k_F^e = (3\pi^2(n/z_e))^{1/3}$ and $k_F^h = (3\pi^2(p/z_h))^{1/3}$.

Now neglecting the possibility that for holes the valleys may be connected to each other (Fig.2 of the main text), we took $z_e = z_h = 3$ and found $k_F^h = k_F^e = 0.82\mathrm{nm}^{-1}$. Depending on the orientation, the actual and anisotropic k_F resides between 0.45 and 2.4nm^{-1} [2]. In this approximation, the mean-free-path can be evaluated using the Drude formula and becomes Supplementary Eq.(2):

$$\ell_0 = \frac{3\pi}{2(z_e + z_h)} \frac{1}{\rho_0} \frac{h}{e^2} \frac{1}{k_F^2} \tag{2}$$

The ℓ_0 values given given in Table 1 of the main text, has been extracted using this equation with $z_e = z_h = 3$. In this approximation, the residual resistivity times the average diameter $\overline{s}$ has a lower boundary set by the carrier concentration (Supplementary Eq.(3)).

$$(\rho_0 \overline{s})_{min} = \frac{3\pi}{2(z_e + z_h)} \frac{h}{e^2} \frac{1}{k_F^2}) \tag{3}$$

Putting $z_e = z_h = 3$, and $n = p = 5.5 \times 10^{19}$ cm^{-3}, one finds $(\rho_0 \overline{s})_{min} = 0.03p\Omega$.m^2. The lowest reported value reported in the scientific literature for a Sb crystal is ($\rho_0 \overline{s} \approx 0.1$p Ω.m^2 [12]), slightly lower than our best Sb crystal (S3) ($\rho_0 \overline{s} = 0.14$p Ω.m^2).

C. Supplementary Note 3 : Dingle mobility

Quantum oscillations have been used to map the Fermi surface of Sb [13]. As seen in Supplementary Figure 3.a, they are easily observable in our crystals. The Dingle analysis yields a mobility, which is much lower than the mobility extracted from residual resistivity. Moreover, as one can see in in Supplementary Figure 3.b, they barely change in four different samples, in spite of their ten-fold variation in residual resistivity. While ℓ_0 in sample S1 is 10 times shorter than in sample S3, the mobility is only 1.2 time larger.

Such a large discrepancy have been found in other dilute metals [6, 14]. In all three cases, the quasi-particle lifetime extracted from transport is orders of magnitude longer than the Dingle scattering time. Our cleanest samples show a 2000-fold discrepancy, which is to be compared to what was reported for the cleanest sample in Cd_2As_3 ($r \approx 10000$) and in WP_2($r \approx 5000$).

The most plausible explanation is to assume that disorder comes with a variety of length scales. There is a broad distribution of the effective size of the scattering centers. The mean-free-path according to residual resistivity is long, because point-like defects (such as extrinsic atoms) do not efficiently scatter a carrier whose wavelength extends over 10 interatomic distances. The mean-free-path according to quantum oscillations is short, because such defects can affect the phase of the travelling electron. They are therefore capable of broadening Landau levels.

This interpretation would also explain the equality of Dingle mobilities in contrast to the difference in residual resistivities. The impurity content of all samples is expected to be identical, because they were grown from an identical melt, but this is not the case of dislocation density and other extended scattering centers, which can be removed by heat treatment.

The amplitude of the magnetoresistance is set by μ_0 extracted from residual resistivity and not by μ_D. The cleaner the sample, the larger its magnetoresistance (see Supplementary Figure 1).

D. Supplementary Note 4 : Low field & low temperature recovery of the Wiedemann-Franz law

Supplementary Figure 4 shows the thermal conductivity plotted as κ/T as a function of T^2 in sample S4 in the low temperature region (where we showed the WF law to be satisfied in the main text) for three different magnetic fields. The arrows point to the value of L_0/ρ_B. We observe that the arrow and y-axis intercept of the linear fit match for the three magnetic fields : the WFL is recovered under the effect of these three fields. Furthermore, the slope of the linear fit to $\kappa/T(T^2)$ remains similar for the different fields. This implies that the magnetic field does not affect the lattice thermal conductivity.

E. Supplementary Note 5 : Thermal conductivity and the third Onsager coefficient

What we have measured is the thermal conductivity measured in absence of charge current. It is to be distinguished from the thermal conductivity measured in absence of electric field, which is a pure diagonal Onsager coefficient [15]. However, in our case, the distinction is totally negligible. The heat current density, J^Q and the particle flow density, J^N are Onsager fluxes responding to Onsager forces : $\nabla \frac{1}{T}$ and $\frac{1}{T}\nabla\mu$ in Supplementary Eq.(4,5).

$$-J^N = L_{11} \frac{1}{T} \nabla \mu + L_{12} \nabla \frac{1}{T} \tag{4}$$

$$J^Q = L_{12}\frac{1}{T}\nabla\mu + L_{22}\nabla\frac{1}{T} \tag{5}$$

The thermal conductivity, κ, in absence of charge current ($J^e = 0$) and the one, κ' in absence of potential gradient ($\nabla\mu = 0$) are to be distinguished. The latter is inversely proportional to the Onsager coefficient L_{22} as shown in Supplementary Eq.(6)

$$\kappa' = \frac{1}{T^2}L_{22} \tag{6}$$

The former is a combination of all three Onsager coefficients and its magnitude is given by Supplementary Eq.(7):

$$\kappa = \kappa'(1 - \frac{S^2\sigma T}{\kappa}) = \kappa'(1 - \frac{S^2}{L}) \tag{7}$$

In our case, since $S < 5\times10^{-6}$V/K and $L \sim L_0 = 2.45\times10^{-8}$ V^2/K^2, one has $\frac{S^2}{L} < 0.001$, implying a negligible difference.

F. Supplementary Note 6 : Ambipolar Thermal Conductivity

The electronic thermal conductivity of a semi-metal includes monopolar contributions from both electrons (κ_e) and holes (κ_h) as well as an ambipolar one associated with electron-hole pairs (κ_{eh}). This last contribution is negligible in Sb at $T \ll T_F$.
Heremans *et al.* showed that the ambipolar contribution to thermal conductivity κ_{eh} can be written as Supplementary Eq.(8) [16]. σ_e and σ_h are respectively the partial electrical conductivities associated with electrons and holes while $E_{F,e}$ and $E_{F,h}$ are the Fermi energies respectively associated with electrons and holes.

$$\kappa_{eh} = (\frac{\pi^2 k_B}{3e})^2 T(\frac{\sigma_e\sigma_h}{\sigma_h+\sigma_e})(\frac{k_BT}{E_{f,h}} + \frac{k_BT}{E_{f,e}})^2 \tag{8}$$

In the temperature range of interest of the present study, $T < 10$K, the Fermi energy of holes and electrons in Sb (featured in Supplementary Table 1) leads to $(k_BT/E_{F,i})^2 \approx 10^{-4}$. This implies, at best, an ambipolar correction to the Lorenz number $L_{eh} = 5.10^{-4}L_0$ at $T = 10$K. Such a correction falls within the experimental error bars of this study and is consequently neglected in our discussion. The small magnitude of the Seebeck coefficient confirms this conclusion.

G. Supplementary Note 7 : Viscosity, thermal conductivity and quasi-particle lifetime in Fermi liquids

Abrikosov and Khatalnikov [17] in their 1959 seminal paper calculated the viscosity of a Fermi liquid given in Supplementary Eq.(9) :

$$\eta T^2 = \frac{64}{45}\frac{\hbar^3 p_F^5}{m^{*4}} < W_\eta > \tag{9}$$

Here $< W_\eta >$ is a temperature-independent parameter representing the angular average of scattering amplitude for viscosity, η, expected to decrease with warming as T^{-2}. The same collisions lead to a thermal conductivity expressed as in Supplementary Eq.(10).

$$\kappa T = \frac{8\pi^2}{3}\frac{\hbar^3 p_F^3}{m^{*4}} < W_\kappa > \tag{10}$$

$< W_\kappa >$, like $< W_\eta >$, is neither dimensionless nor universal. The amplitude of both depends on the strength and the anisotropy of interaction and, in the case of ^{3}He, strongly depends on the spin components of the overlapping wave-functions. Numerous experiments confirmed that $\eta \propto T^{-2}$ [18–20] and $\kappa \propto T^{-1}$ [21, 22]. In the case of thermal conductivity, the most elaborate set of measurements performed by Greywall [22] found that at zero pressure, the asymptotic value for κT is $\kappa T|_0 = 2.9\times10^{-4}$W.m^{-1}. This is about 0.6 of the theoretical value of calculated by Brooker and Sykes (5×10^{-4}W.m^{-1}) [23].

Calkoen and van Weert [24] showed that in the zero temperature limit, one can write Supplementary Eq.(11).

$$\kappa T|_0 = \frac{5}{18\pi^3}\frac{p_F^3 v_F^2}{A^2} \tag{11}$$

In this equation, the notation takes $\hbar = 1$. In our equation 2 of the main text, in order to enhance clarity, we have introduced the dimensionless parameter B_0, which is simply proportional to A^2 as shown in Supplementary Eq.(12).

$$B_0 = \frac{9\pi^3 A^2}{10\hbar^2} \tag{12}$$

Calkoen and van Weert [24] found that in ^{3}He, a nearly ferromagnetic liquid, the magnitude of A and its variation with pressure is compatible with the Landau parameters extracted from specific heat data [25].

The fundamental reason behind the temperature dependence of η and κ is the quadratic temperature dependence of the relaxation time, which can be written as Supplementary Eq.(13) [26]:

$$\frac{\hbar}{\tau_{qp}} = \frac{(\pi k_B T)^2}{32 E_F} < A >_{\theta,\phi} \tag{13}$$

Here $< A >_{\theta,\phi}$ represents the angular averages of quasi-particle scattering amplitudes for transition between spin singlet and spin triplet states [26]. In the case of ^{3}He, measurements of viscosity [19] and thermal conductivity [22] have found values for $\tau_\kappa T^2$ and $\tau_\eta T^2$ close to each other. $\tau_\kappa T^2$ can be extracted from the heat capacity per volume C_v, using Supplementary Eq.(14) :

$$\tau_\kappa = 3\frac{\kappa}{C_v v_F^2} \tag{14}$$

As in the case of ^{3}He, we have used the electronic specific heat of Sb ($\gamma = 0.105$mJ.mol^{-1}.K^{-2}) [27] and the average Fermi velocity to calculate $\tau_\kappa T^2$ in Sb.

Supplementary References

1. Issi, J. Low temperature transport properties of the group V semimetals. *Australian Journal of Physics* **32,** 585–628 (1979).
2. Liu, Y. & Allen, R. E. Electronic structure of the semimetals Bi and Sb. *Physical Review B* **52,** 1566–1577 (1995).
3. Edelman, V. S. Electrons in bismuth. *Advances in Physics* **25,** 555–613 (1976).
4. Zhu, Z. *et al.* Quantum oscillations, thermoelectric coefficients, and the Fermi surface of semimetallic WTe_2. *Physical Review Letters* **114,** 176601 (2015).
5. Schönemann, R. *et al.* Fermi surface of the Weyl type-II metallic candidate WP_2. *Physical Review B* **96,** 121108 (2017).
6. Kumar, N. *et al.* Extremely high magnetoresistance and conductivity in the type-II Weyl semimetals WP_2 and MoP_2. *Nature Communications* **8,** 1642 (2017).
7. Jaoui, A. *et al.* Departure from the Wiedemann–Franz law in WP_2 driven by mismatch in T-square resistivity prefactors. *npj Quantum Materials* **3,** 64 (2018).
8. Fauqué, B. *et al.* Magnetoresistance of semimetals: the case of antimony. *Physical Review Materials* **2,** 114201 (2018).
9. Collignon, C., Lin, X., Rischau, C. W., Fauqué, B. & Behnia, K. Metallicity and superconductivity in doped strontium titanate. *Annual Review of Condensed Matter Physics* **10,** 25–44 (2019).
10. Tsujii, N., Yoshimura, K. & Kosuge, K. Deviation from the Kadowaki–Woods relation in Yb-based intermediate-valence systems. *Journal of Physics: Condensed Matter* **15,** 1993 (2003).
11. Wang, J. *et al.* T-square resistivity without umklapp scattering in dilute metallic Bi_2O_2Se. *Nature Communications* **11,** 3846 (2020).
12. Hatzopoulos, Z. & Aubrey, J. E. Size effects in the electrical resistivity and mean transverse electric field ratio of bismuth and antimony. *Journal of Physics F: Metal Physics* **15,** 1093–1101 (1985).
13. Herrod, R., Gage, C. & Goodrich, R. Fermi surface of antimony: radio-frequency size zffect. *Physical Review B* **4,** 1033 (1971).
14. Liang, T. *et al.* Ultrahigh mobility and giant magnetoresistance in the Dirac semimetal Cd_3As_2. *Nature Materials* **14,** 280–284 (2015).
15. Behnia, K. *Fundamentals of Thermoelectricity* (Oxford University Press, 2015).
16. Heremans, J., Issi, J.-P., Rashid, A. & Saunders, G. Electrical and thermal transport properties of arsenic. *Journal of Physics C: Solid State Physics* **10,** 4511 (1977).
17. Abrikosov, A. A. & Khalatnikov, I. M. The theory of a Fermi liquid (the properties of liquid ^{3}He at low temperatures). *Reports on Progress in Physics* **22,** 329–367 (1959).
18. Black, M. A., Hall, H. E. & Thompson, K. The viscosity of liquid helium 3. *Journal of Physics C: Solid State Physics* **4,** 129–142 (1971).
19. Bertinat, M. P., Betts, D. S., Brewer, D. F. & Butterworth, G. J. Damping of torsional oscillations of a quartz crystal cylinder in liquid helium at low temperatures. I. Viscosity of pure ^{3}He. *Journal of Low Temperature Physics* **16,** 479–494 (1974).

20. Alvesalo, T. A., Collan, H. K., Loponen, M. T., Lounasmaa, O. V. & Veuro, M. C. The viscosity and some related properties of liquid ^{3}He at the melting curve between 1 and 100 mK. *Journal of Low Temperature Physics* **19,** 1–37 (1975).
21. Abel, W. R., Johnson, R. T., Wheatley, J. C. & Zimmermann, W. Thermal conductivity of pure He^3 and of dilute solutions of He^3 in He^4 at low temperatures. *Physical Review Letters* **18,** 737–740 (1967).
22. Greywall, D. S. Thermal conductivity of normal liquid ^{3}He. *Physical Review B* **29,** 4933 (1984).
23. Brooker, G. A. & Sykes, J. Transport Properties of a Fermi liquid. *Physical Review Letters* **21,** 279–282 (1968).
24. Calkoen, C. J. & van Weert, C. G. Thermal conductivity of normal liquid ^{3}He at finite temperatures. *Journal of Low Temperature Physics* **64,** 429–440 (1986).
25. Greywall, D. S. Specific heat of normal liquid ^{3}He. *Physical Review B* **27,** 2747–2766 (1983).
26. Wolfle, P. Low-temperature properties of liquid ^{3}He. *Reports on Progress in Physics* **42,** 269–346 (1979).
27. McCollum, D. C. & Taylor, W. A. Low-temperature specific heat of antimony. *Physical Review* **156,** 782–784 (1967).
